# Molecular characteristics of *isocitrate dehydrogenase 1* R132C–mutant diffuse gliomas: association with *TP53* alterations and Li-Fraumeni syndrome

**DOI:** 10.1186/s40478-026-02369-w

**Published:** 2026-07-03

**Authors:** Shinji Yamashita, Fumitaka Matsumoto, Kiyotaka Saito, Masato Hidaka, Souma Arikawa, Tomoki Kawano, Tomohiro Kawano, Mitsuru Tamura, Hironobu Okuyama, Nayuta Higa, Ryosuke Hanaya, Toshiaki Akahane, Akihide Tanimoto, Yuichiro Sato, Yoshiko Okita

**Affiliations:** 1 Department of Neurosurgery, Division of Clinical Neuroscience, Faculty of Medicine, University of Miyazaki, 5200 Kihara Kiyotake, Miyazaki, 889-1692 Japan; 2 Department of Neurosurgery, Kagoshima University Graduate School of Medical and Dental Sciences, Kagoshima, Japan; 3 Department of Pathology, Kagoshima University Graduate School of Medical and Dental Sciences, Kagoshima, Japan; 4 Section of Oncopathology and Morphological Pathology, Department of Pathology, Faculty of Medicine, University of Miyazaki, Miyazaki, Japan

**Keywords:** Low-grade glioma, *Isocitrate dehydrogenase 1* R132C-mutant, Li-Fraumeni syndrome

## Abstract

**Background:**

The non-canonical *isocitrate dehydrogenase 1* (*IDH1*) R132C mutation is rare in diffuse gliomas but appears to be enriched in tumors associated with Li-Fraumeni syndrome (LFS), which is caused by germline tumor protein p53 (*TP53*) mutations. However, the molecular relationship between *TP53* alterations and *IDH1* R132C, as well as the prevalence of LFS among patients with R132C–mutant gliomas, remains unclear.

**Methods:**

We analyzed 93 consecutive patients retrospectively with *IDH1*–mutant diffuse gliomas (Central Nervous System World Health Organization (CNS WHO) Grades 2 and 3) treated at our institution between 2005 and 2025. *IDH, TP53*, and *ATRX* status were assessed by immunohistochemistry, Sanger sequencing, multiplex ligation-dependent probe amplification, and targeted next-generation sequencing. Germline *TP53* mutations were assessed using peripheral blood samples. DNA methylation profiling was performed using the Methylscape platform and the DKFZ methylation classifier.

**Results:**

Three of 90 *IDH1*-mutant gliomas (3%) harbored the R132C variant. All three tumors were astrocytomas with concurrent *TP53* mutation, *ATRX* alteration, and 1p/19q non-codeletion. Exploratory cancer cell fraction analysis suggested that both *IDH1* R132C and *TP53* mutations were present in the major tumor cell population, supporting a biological association between *TP53* alterations and the occurrence of the *IDH1* R132C variant. One patient harbored a pathogenic germline *TP53* mutation despite lacking a family history or fulfilling the established clinical diagnostic criteria for LFS. DNA methylation analysis demonstrated partial overlap with conventional *IDH*–mutant astrocytoma and also revealed atypical clustering patterns.

**Conclusions:**

*IDH1* R132C-mutant gliomas are characterized by concurrent *TP53* mutation, *ATRX* alteration, and 1p/19q non-codeletion. Although based on a small cohort, our findings support a biological association between *TP53* alterations and the rare *IDH1* R132C variant and suggest that germline *TP53* testing may be considered in selected patients, particularly younger individuals, to identify previously unrecognized Li-Fraumeni syndrome.

**Supplementary Information:**

The online version contains supplementary material available at https://doi.org/10.1186/s40478-026-02369-w.

## Introduction

Isocitrate dehydrogenase (*IDH*) mutations define a molecular subset of adult-type diffuse gliomas characterized by a relatively favorable prognosis and a distinct pathogenesis. Most *IDH*–mutant gliomas harbor a substitution of arginine to histidine at codon 132 (*IDH1* R132H). However, approximately 10% of cases exhibit non-canonical *IDH* mutations, including non-R132H *IDH1* or *IDH2* mutations [1]. Reported frequencies of non-R132H *IDH1* variants include R132C (2.9–4.3% of *IDH1*–mutant cases), R132G (1.0–2.5%), R132S (1.1–2.2%), and R132L (0.2–0.6%) [1–5].

Among these non-canonical *IDH1* variants, the R132C mutation has attracted particular attention due to its apparent enrichment in gliomas arising in Li-Fraumeni syndrome (LFS), a hereditary cancer predisposition syndrome caused by germline *TP53* mutations [5]. Previous reports have indicated that gliomas in patients with LFS more frequently harbor the *IDH1* R132C mutation rather than the canonical R132H variant, suggesting a potential biological association between *TP53* dysfunction and this specific substitution [5–7]. However, the mechanistic relationship between *TP53* alterations and the *IDH1* R132C mutation in gliomagenesis remains unclear.

In this study, we investigated the mutational landscape of glioma-related genes in *IDH1* R132C-mutant gliomas using a targeted next-generation sequencing (NGS) panel. We also evaluated germline *TP53* mutation status to explore the potential link between *IDH1* R132C and LFS. Furthermore, we performed DNA methylation-based classification of *IDH1* R132C gliomas, comparing these tumors with diffuse astrocytoma harboring canonical *IDH1* mutations.

## Materials and methods

### Study population and clinical data

In this study, we collected data from 93 consecutive, unselected patients with *IDH*–mutant gliomas (Central Nervous System World Health Organization [CNS WHO] Grades 2 and 3) who were treated at Miyazaki University Hospital between April 2005 and February 2025. Clinical data, including age at diagnosis, sex, histological type, and date of death or last follow-up, were retrospectively obtained from the electronic medical records. This retrospective study was approved by the Institutional Review Board of Miyazaki University Hospital, Japan (Approval No. G-1135).

### Laboratory analyses

*IDH* mutation status was assessed by immunohistochemical analysis using a specific R132H antibody and by direct sequencing.

Immunostaining was performed according to the manufacturer’s protocol. Briefly, formalin-fixed paraffin-embedded (FFPE) tissue samples were sectioned at 2-μm thickness, deparaffinized in xylene, and rehydrated in graded ethanol. Antigen retrieval was performed by steaming with citric acid buffer (pH 6.0) for 20 min using an autoclave. Sections were incubated overnight at 4 °C with a monoclonal anti-human IDH1-R132H antibody (1:20 dilution; clone H09; Dianova, Hamburg, Germany). A definite cytoplasmic immunoreaction product was scored as positive cells. Samples with more than 10% positively stained tumor cells were classified as *IDH1*–mutant.

Direct DNA sequencing was conducted as previously described. Genomic DNA was extracted from frozen tissue samples using the QIAamp DNA Mini Kit (Qiagen, Valencia, CA). For *IDH1* analysis, a 90-base pair (bp) fragment was amplified using the sense primer *IDH1* (forward: 5’-GGCTTGTGAGTGGATGGGTA-3’) and the antisense primer *IDH1* (reverse: 5’-GCAAAATCACATTATTGCCAAC-3’). For *IDH2* analysis, a 102-bp fragment was amplified using the sense primer *IDH2* (forward: 5’-AGCCCATCATCTGCAAAAAC-3’) and the antisense primer *IDH2* (reverse: 5’-CTCCACCCTGGCCTACCT-3’). Polymerase chain reaction (PCR) was performed in a 25-μL reaction mixture containing 50-ng tumor genomic DNA, 1 μL of each forward and reverse primer (10 μM), and 12.5-μL of GoTaq Hot Start Green Master Mix (Promega, Fitchburg, WI, USA). Sequence reactions were performed using the Big Dye Terminator v1.1 Cycle Sequencing Kit (Thermo-Fisher Scientific, Waltham, MA, USA) in a 20-μL reaction mixture containing 1-μL of PCR product, 4-μL of sequencing buffer, 2-μL of forward primer (5 μM), and 1-μL of Big Dye Terminator Ready Reaction Mix. The sequencing products were analyzed using an ABI3500 Genetic Analyzer (Thermo-Fisher Scientific).

Chromosome 1p/19q codeletion status was assessed using fluorescence in situ hybridization (FISH) or PCR-based microsatellite marker analysis [8, 9]. FISH analysis for 1p/19q loss of heterozygosity was performed on 5-μm FFPE tissue sections using dual-probe hybridization with Vysis LSI FISH probes, according to the manufacturer’s instructions (Abbott Japan Co. Ltd., Tokyo, Japan). Dual-color probe sets targeting 1p36/1q25 and 19q13/19p13 were used for locus-specific 1p and 19q analyses, respectively. Nuclei were counterstained with 4,6-diamidino-2 phenylindole. A cutoff value of 20% of nuclei showing deletion signals was used to define loss of heterozygosity.

Microsatellite analysis was performed on paired tumor and peripheral blood DNA samples using the following 19 polymorphic markers: 11 markers on Chromosome 1p (1p11—36.22; D1S1166, D1S495, D1S207, D1S435, D1S2835, D1S2797, D1S2892, D1S2657, D1S2647, D1S402, and D1S244) and 7 markers on Chromosome 19q (19q12—13.33; D19S596, D19S412, D19S112, D19S219, D19S420, D19S220, and D19S919). PCR amplification was performed, and the products were analyzed by capillary gel electrophoresis using a GeneMapper system on an ABI 3730 Genetic Analyzer (Applied Biosystems). Patient-matched germline DNA from a peripheral blood sample was used as a control.

*IDH* mutations, Chromosome 1p/19q codeletion status, and copy number of *CDKN2A/B* were also evaluated using multiplex ligation-dependent probe amplification (MLPA). Genomic DNA was extracted from fresh-frozen tissue samples using standard protocols. MLPA was conducted using the SALSA MLPA Probemix P088 Glioma-2 and P096 1p/19q probe sets (MRC-Holland, Amsterdam, The Netherlands) according to the manufacturer’s instructions. Briefly, 100 ng of genomic DNA was denatured and hybridized with the probe mix at 60 °C for 16 h, followed by ligation and PCR amplification using MLPA-specific primers. The amplified fragments were separated by capillary electrophoresis on an ABI 3500 Genetic Analyzer (Applied Biosystems), and peak areas were quantified using Coffalyser.Net software (MRC-Holland). Relative copy number ratios were calculated by normalization to internal reference probes and control DNA samples (human genomic DNA female and male, Promega, Madison, WI, USA). Copy number loss or gain was defined according to established MLPA ratio thresholds.

Immunohistochemistry staining for TP53 and ATRX was performed using the same protocol as for IDH1 R132H immunostaining. The following primary antibodies were used: monoclonal mouse anti-human TP53 (1:300 dilution; clone DO-7; Genetex, Irvine, CA, USA) and monoclonal mouse anti-human ATRX (1:400 dilution; clone AMab-6; Fujifilm Wako, Osaka, Japan).

The nucleotide sequences of *TP53* exons 5–8 were determined by direct (Sanger) sequencing, as previously described [10]. Briefly, PCR amplification was performed in a total volume of 25 µL containing 20 ng of genomic DNA, 1 µL each of forward and reverse primers (10 µM), 12.5 µL of GoTaq Hot Start Green Master Mix (Promega, Tokyo, Japan), and deionized water. The PCR conditions were as follows: initial denaturation at 94 °C for 5 min; 35 cycles of denaturation at 94 °C for 50 s, annealing at 60 °C for 1 min, and extension at 72 °C for 70 s; followed by a final extension at 72 °C for 5 min. For exon 5, the annealing temperature was adjusted to 55 °C. PCR products corresponding to exons 5–8 were purified using ExoSAP-IT (Thermo-Fisher Scientific, Tokyo, Japan). Cycle sequencing was performed using the BigDye Terminator v3.1 Cycle Sequencing Kit (Thermo-Fisher Scientific), and the products were analyzed using an ABI PRISM 3130xl Genetic Analyzer (Thermo-Fisher Scientific). Sequence data were compared with the *TP53* reference sequence (NG_017013.2) from the National Center for Biotechnology Information database.

### Targeted NGS

Targeted NGS was performed using a custom-designed gene panel developed at Kagoshima University, as previously described [11, 12]. The panel covers 51 glioma-associated genes, including *IDH1, IDH2, TP53, ATRX, EGFR, PDGFRA, CDKN2A/B*, and the *TERT* promoter, as well as genomic regions enabling assessment of 1p/19q codeletion status.

Genomic DNA was extracted from formalin-fixed paraffin-embedded (FFPE) tumor tissues. Targeted libraries were prepared using the GeneRead DNAseq Panel (Qiagen, Hilden, Germany) according to the manufacturer’s protocol and sequenced on an Illumina NextSeq 1000 platform (Illumina, San Diego, CA, USA) using paired-end sequencing (2 × 150 bp).

The mean on-target sequencing depth ranged from 709× to 1182× across the three IDH1 R132C-mutant cases, with more than 95% of target bases covered at a depth of at least 200× . Raw FASTQ files were processed using the Qiagen Web Portal pipeline. Sequence reads were aligned to the human reference genome (GRCh37/hg19) using a BWA-based aligner. A commercial bioinformatics analysis service (Mitsubishi Electric Software, Hyogo, Japan) was used to interpret the sequence results. Tumor purity was estimated using the Qiagen CNV analysis module. Variant allele frequency (VAF) was calculated as the proportion of variant-supporting reads among all high-quality reads covering the genomic locus. Variants were identified using smCounter2 with default parameters, and only variants with a VAF ≥ 10% were included in the analysis.

To estimate the cancer cell fraction (CCF) of *IDH1* and *TP53* mutations, observed variant allele frequency (VAF), tumor purity, and total gene copy number obtained from targeted next-generation sequencing were incorporated into a purity- and copy number-adjusted model for somatic mutation clonality, as previously described with a simplified modification[13, 14].

CCF was estimated according to the following equation:

$$ {\mathrm{CCF}} = \frac{{VAF \times \left[ {\left( {1 - p} \right) \times 2 + p \times CN} \right]}}{p \times m} $$

where *p* represents tumor purity, *CN* is the total copy number of the locus, and *m* is the estimated number of mutated alleles.

Because allele-specific copy number analysis was not performed, the mutant allele copy number could not be directly determined. Therefore, one copy of the wild-type allele was assumed, and the mutant allele copy number was estimated as:

*m* = CN − 1.

This simplified model assumes that any additional copies are derived from the mutant allele. Accordingly, the estimated CCF values should be regarded as exploratory and interpreted with caution.

### DNA methylation analysis

Genome-wide DNA methylation profiling was conducted using the Illumina Infinium methylation array following the manufacturer’s instructions. DNA was extracted from fresh-frozen tumor tissue samples and subjected to bisulfite conversion prior to array hybridization. Methylation data were analyzed using a DNA methylation-based tumor classification strategy. For dimensionality reduction and visualization, t-distributed stochastic neighbor embedding analysis was conducted using the Methylscape platform in conjunction with the Bethesda classifier v2 (accessed October 17, 2025; https://methylscape.org) and the DKFZ methylation classifier (version 12.8; accessed October 17, 2025; https://www.molecularneuropathology.org). Methylation profiles of the present cases were compared with reference datasets from each platform to assess their clustering patterns and methylation class assignments.

## Results

### In-house analysis of *IDH1* R132C cases

Between April 2006 and February 2025, a total of 93 patients with *IDH*–mutant gliomas underwent surgical resection and histopathological diagnosis at Miyazaki University Hospital. The median at diagnosis age was 47 years, and the cohort included 44 men and 49 women. Ninety cases (97%) harbored *IDH1* mutations, whereas three cases (3%) harbored *IDH2* mutations. Among the IDH1-mutant cases, 87 (97%) harbored the canonical R132H mutation, whereas 3 (3%) harbored the non-canonical R132C mutation, as determined by direct sequencing or MLPA (Table 1).

**Table 1:** Patient characteristics

Parameter (n)	No. of patient	%
Age (93)		
Median	47	
Range	16–79	
Sex (93)		
Male	44	47
Female	49	53
IDH mutation (93)		
IDH1	90	97
IDH2	3	3
IDH1 mutation subtype (90)		
R132H	87	97
R132C	3	3
1p/19q status (93)		
Wild	66	71
LOH	27	29
CDKN2A/B (18)		
Wild	14	78
Deletion	4	22

All three *IDH1* R132C–mutant cases were 1p/19q non-codeleted and demonstrated TP53 positivity and ATRX loss by immunohistochemistry. Morphologically, no mitotic figures or necrosis were observed, and all tumors were classified as astrocytoma, *IDH*–mutant, CNS WHO Grade 2. The tumors were located at the frontal lobe in two cases and at the brainstem in one case. During a mean follow-up period of 53 months, none of the patients experienced recurrence (Table 2).

**Table 2:** Characteristics of IDH1 R132C mutated cases

No	Age	Sex	Tumor location	IDH1 mutation	1p/19q LOH	TP53 IHC	ATRX IHC	Pathological diagnosis CNS WHO grade	PFS (months)	OS (months)
1	46	M	Frontal	p.R132C	intact	Positive	loss: (−)	Astrocytoma, IDH mutant Grade 2	121	121
2	31	M	Brain stem	p.R132C	intact	Positive	loss: (−)	Astrocytoma, IDH mutant Grade 2	31	31
3	22	F	Frontal	p.R132C	intact	Positive	loss: (−)	Astrocytoma, IDH mutant Grade 2	10	10

### Genetic analysis using a panel sequencing approach

Targeted NGS panel analysis was subsequently performed on cases harboring the *IDH1* R132C mutation. As expected, *IDH1* R132C, *TP53*, and *ATRX* mutations were detected in all three cases. To further characterize the clonal architecture of *IDH1* and *TP53* mutations, cancer cell fraction (CCF) was estimated using tumor purity, observed variant allele frequency (VAF), and total gene copy number, assuming one copy of the wild-type allele (Table 3; Supplementary Fig. 1–9). Under this assumption, the estimated CCF values for *IDH1* were 0.94, 1.01, and 0.84 in the three cases, indicating that the *IDH1* mutations were clonal events present in nearly all tumor cells. In contrast, the estimated CCF values for *TP53* were 1.89, 0.99, and 1.65. Although the estimated CCF values exceeding 1 are biologically implausible and may reflect copy-neutral loss of heterozygosity (cnLOH) or other allele-specific copy number alterations, these findings suggest that TP53 mutations were represented in the major tumor cell population, similar to IDH1 R132C mutations. Collectively, these findings indicate that TP53 mutations were consistently represented at a frequency comparable to that of the clonal IDH1 R132C mutation in all three tumors.

**Table 3:** Results of NGS panel sequence

No	IDH1				TP53				ATRX				Tumor purity (%)
	Mutation	VAF (%)	CN	Estimated CCF*	Mutation	VAF (%)	CN	Estimated CCF*	Mutation	VAF (%)	CN	Estimated CCF*	
1	p.R132C	40.4	2.4	0.94	p.R273C	70.4	2.1	1.89	p.A279_C280ins	68.3	2.5	1.53	70
2	p.R132C	50.7	2.5	1.01	p.R267W	55.4	2.9	0.99	p.K307Ifs	74.2	2.4	1.80	80
3	p.R132C	31.1	2.1	0.84	p.R282P	68	2.3	1.65	p.D436Vfs	28	1.9	0.86	70

### Germline *TP53* mutation analysis and diagnosis of LFS

**Fig. 1:**
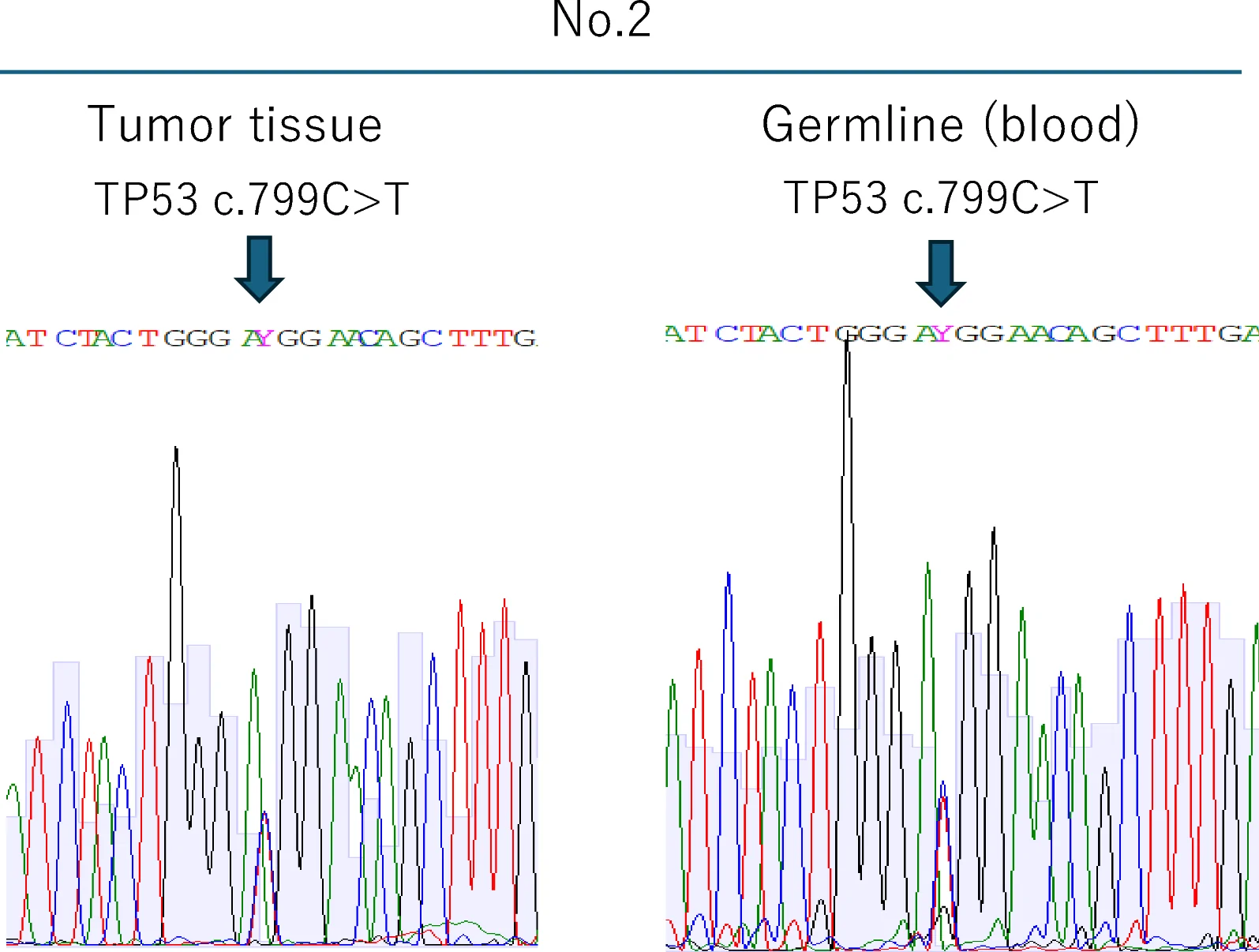
Germline *TP53* mutation. *TP53* mutation analysis in Case 2. The identical *TP53* c.799C > T mutation was identified in both the tumor and peripheral blood, confirming a germline mutation compatible with Li-Fraumeni syndrome

Analysis of germline *TP53* mutation using peripheral blood revealed that one of the three patients (33%) harbored the same *TP53* mutation as that identified in the tumor (Fig. 1; Supplementary Fig. 10). This variant was classified as pathogenic in ClinVar, leading to the diagnosis of LFS (Supplementary Fig. 11). Notably, this patient (Case 2) had no personal or family history of cancer, fulfilling the clinical diagnostic or Chompret criteria for LFS (Supplementary Fig. 12). The tumor in this case was primarily located in the brainstem and appeared as a hyperintense lesion on fluid-attenuated inversion recovery magnetic resonance imaging. Histopathological and molecular analyses established a diagnosis of astrocytoma, *IDH*–mutant, CNS WHO Grade 2 (Supplementary Figs. 13–15).

### DNA methylation classification

**Fig. 2:**
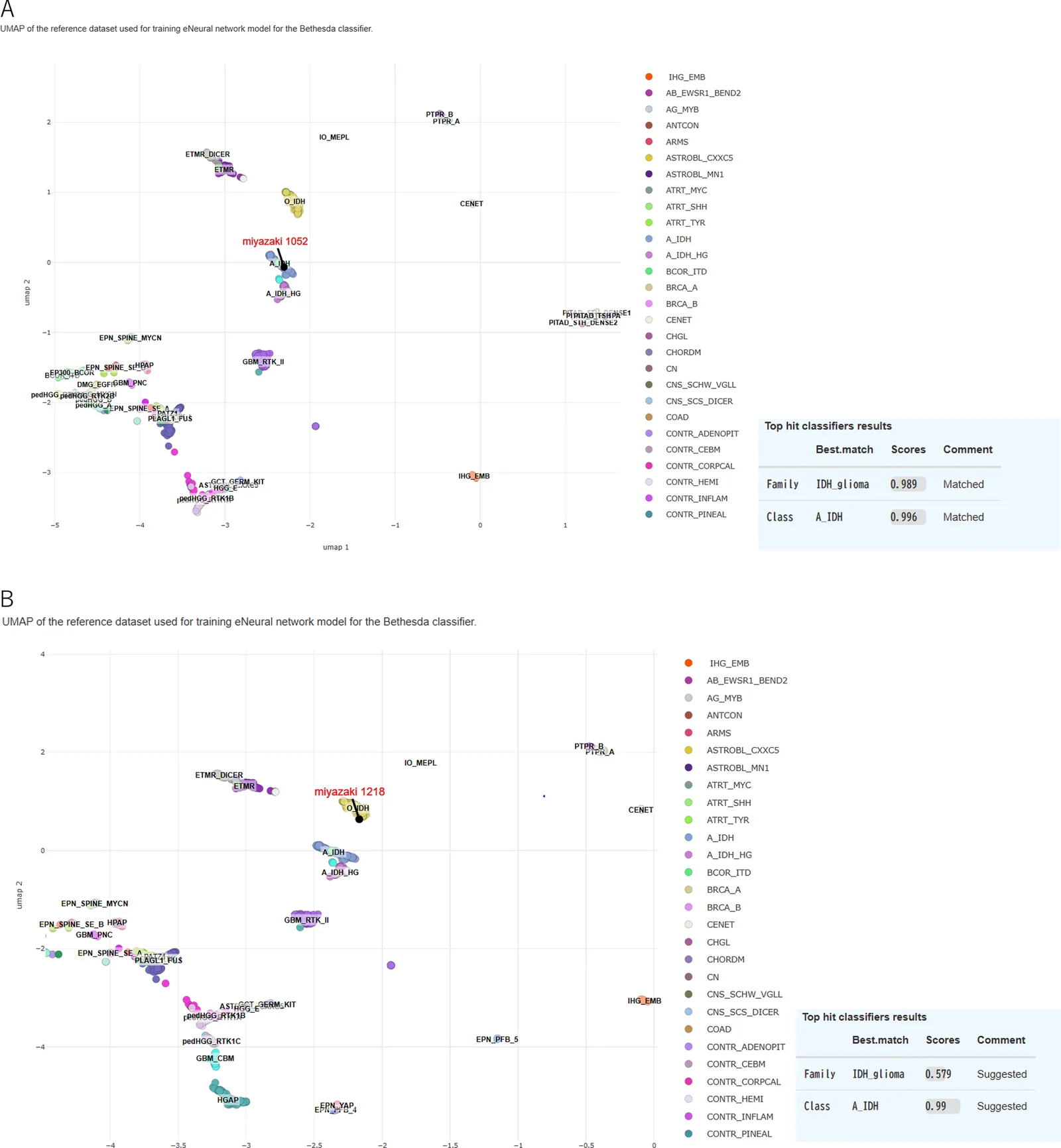
DNA methylation classification in *IDH1* R132C–mutant gliomas. **A** DNA methylation classification of Case 2. Methylscape classified the tumor as *IDH*–mutant glioma, A-IDH. **B** DNA methylation classification of Case 3. Methylscape indicated a suspected *IDH*–mutant glioma, A-IDH classification

DNA methylation profiling was additionally performed. In Case 2, the Methylscape analysis classified the tumor as an *IDH*–mutant glioma, A-IDH. In contrast, the DKFZ methylation classifier categorized the tumor as diffuse glioma, *IDH*–mutant, without a definitive match to either the astroglial or oligodendroglial subclass (Fig. 2A; Supplementary Fig. 16). In Case 3, Methylscape analysis yielded only a tentative classification of IDH–mutant glioma, A-IDH, in contrast to Case 2, which showed a definitive match to this class. Similarly, the DKFZ methylation classifier assigned this tumor to diffuse glioma, *IDH*–mutant, but again without a clear match to either the astroglial or oligodendroglial subclass (Fig. 2B; Supplementary Fig. 17).

## Discussion

In our cohort, three of 90 *IDH1*–mutant gliomas (3%) harbored the R132C variant, a frequency consistent with previous reports (2.9–4.3%) [1–5]. The median age of patients with *IDH1* R132C–mutant tumors was 31 years, which is notably younger than that of the overall *IDH*–mutant cohort (47 years). Hartmann et al. previously reported that R132C–mutant gliomas occur at significantly younger ages compared with tumors harboring canonical *IDH1* mutations, and our findings are consistent with this observation [15]. Poetsch et al. also reported that gliomas harboring non-canonical *IDH* mutations were associated with a more favorable prognosis than those with canonical *IDH1* mutations [16]. Although none of our three patients experienced tumor recurrence during a median follow-up of 53 months, the small number of cases and relatively limited follow-up preclude any conclusions regarding the prognostic significance of the *IDH1* R132C mutation.

At our institution, *IDH* mutation screening is routinely performed using MLPA with a 1p/19q probe set. This approach enables the detection of non-canonical mutations, such as *IDH1* R132C and *IDH2* R172K/M, which cannot be identified by *IDH1* R132H-specific immunohistochemistry and was therefore useful in the present study [16]. However, MLPA is limited in its ability to detect rare variants outside these targeted loci. Consequently, Sanger sequencing remains the preferred method for comprehensive *IDH* mutation analysis, while NGS panels provide the most comprehensive and sensitive approach when available.

Several reports have revealed that gliomas in patients with LFS frequently, and in some series exclusively, harbor the *IDH1* R132C mutation [5, 7]. Given that *IDH1* R132C accounts for only approximately 3%–4% of sporadic *IDH1*–mutant gliomas, this enrichment suggests a potential biological association. Watanabe et al. hypothesized that the R132C variant may arise preferentially when the *TP53* mutation occurs prior to the *IDH1* alteration [5]. Based on our estimated cancer cell fraction (CCF) analysis, TP53 mutations were present at a frequency comparable to that of the clonal IDH1 R132C mutation in all three cases, suggesting that TP53 alterations were distributed throughout nearly the entire tumor cell population. Although these findings do not establish the temporal sequence of mutational events, they provide supportive evidence for Watanabe’s hypothesis that TP53 dysfunction may establish a molecular context that favors the emergence of the IDH1 R132C substitution [5]. Interestingly, Hayes et al. reported a multicentric low-grade glioma case with a germline *TP53* mutation in which one tumor harbored *IDH1* R132C while another harbored *IDH1* R132H [6]. Thus, in the presence of a germline *TP53* mutation, the R132C variant is not invariably generated, implying that additional microenvironmental or stochastic factors may influence the specific *IDH1* mutation outcomes. Further analyses are warranted to clarify these mechanisms.

Although several studies have examined the frequency of R132C mutations among LFS-associated gliomas, to our knowledge no study has evaluated the reverse relationship, namely, the prevalence of LFS among patients with *IDH1* R132C–mutant gliomas [5, 7]. In our series, one of three patients with *IDH1* R132C–mutant gliomas harbored a germline *TP53* mutation. Notably, this patient, like the other two, had no family history suggestive of LFS and did not meet the classical clinical diagnostic criteria or Chompret criteria [17, 18]. A nationwide Japanese study of LFS pedigrees reported that 33.3% of families did not meet established clinical criteria and that de novo mutations accounted for 25% of cases [19, 20]. These findings underscore the limitations of relying solely on clinical criteria for the diagnosis of LFS. Although the present study was limited to only three cases and requires validation in larger independent cohorts, our findings suggest that previously unrecognized LFS may be present in a subset of patients with *IDH1* R132C-mutant gliomas. Therefore, consideration of germline *TP53* testing may be warranted, particularly in younger patients or those with atypical clinical features, to facilitate the identification of underlying LFS and enable appropriate surveillance, genetic counseling, and risk assessment for affected patients and their families.

As reported by Hayes et al. and Sloan et al., gliomas harboring the canonical *IDH1* R132H mutation can also occur in patients with germline *TP53* mutations [6, 7]. In our cohort, however, germline *TP53* testing was not performed in the 87 patients with *IDH1* R132H-mutant gliomas. Therefore, the prevalence of germline *TP53* mutations in this subgroup remains unknown, and a direct comparison with patients harboring *IDH1* R132C-mutant gliomas could not be performed. To our knowledge, no previous study has systematically evaluated the prevalence of germline *TP53* mutations among patients with *IDH1* R132H-mutant gliomas. Nevertheless, accumulating evidence indicates that the *IDH1* R132C variant is markedly enriched in gliomas arising in the setting of Li-Fraumeni syndrome [5, 6].

To compare the DNA methylation profiles of R132C–mutant cases with those of astrocytomas harboring the canonical *IDH* mutations, we conducted the analysis using Methylscape (NIH) and the CNS Tumor Methylation Classifier v12.8 (DKFZ). Methylscape analysis showed that Case 2 clustered with conventional *IDH*–mutant astrocytoma; however, Case 3 did not cluster with this group. Similarly, in the DKFZ methylation classifier analyses, both Cases 2 and 3 were classified as diffuse glioma, *IDH*–mutant, but neither showed a definitive match to the astrocytoma subclass. Mircea et al. reported that genome-wide DNA methylation levels are significantly higher in non-canonical *IDH*–mutant tumors than in *IDH1* R132H tumors, which may contribute to transcriptional repression genes and a more favorable prognosis [21]. In *IDH1* R132C–mutant cases, globally increased methylation levels may lead to subtle alterations in methylation patterns, potentially leading to a distinct cluster profile. Further studies with larger cohorts are warranted to validate these findings.

This study has several limitations. First, only three *IDH1* R132C-mutant gliomas were identified, limiting the statistical power and generalizability of our findings. Second, because allele-specific copy number analysis was not performed, the estimated cancer cell fraction (CCF) values were based on the assumption of one wild-type allele and should therefore be regarded as exploratory. Third, systematic germline *TP53* testing was not available for the 87 patients with *IDH1* R132H-mutant gliomas, precluding direct comparison of the prevalence of germline *TP53* mutations between canonical and non-canonical *IDH1*-mutant tumors. Therefore, the present findings should be considered hypothesis-generating and require validation in larger independent cohorts.

## Conclusions

*IDH1* R132C-mutant gliomas were characterized by concurrent *TP53* mutation, *ATRX* alteration, and 1p/19q non-codeletion. Exploratory cancer cell fraction analysis supported a biological association between *TP53* alterations and the occurrence of the rare *IDH1* R132C variant. In addition, one of the three patients harbored a pathogenic germline *TP53* mutation despite lacking clinical features of Li-Fraumeni syndrome. Although validation in larger cohorts is required, germline *TP53* testing may be considered in selected patients with *IDH1* R132C-mutant gliomas, particularly younger individuals. These findings provide new insight into the molecular background of *IDH1* R132C-mutant gliomas and their potential relationship with Li-Fraumeni syndrome.

## Supplementary Information

Additional file1 (PPTX 13628 kb)

## Data Availability

No datasets were generated or analysed during the current study.
